# The Peptidyl-prolyl Isomerase Pin1 in Neuronal Signaling: from Neurodevelopment to Neurodegeneration

**DOI:** 10.1007/s12035-020-02179-8

**Published:** 2020-10-21

**Authors:** Francesca Fagiani, Stefano Govoni, Marco Racchi, Cristina Lanni

**Affiliations:** 1 Department of Drug Sciences, Pharmacology Section, University of Pavia, Viale Taramelli 14, 27100 Pavia, Italy; 2 Scuola Universitaria Superiore IUSS Pavia, P.zza Vittoria, 15, 27100 Pavia, Italy

**Keywords:** Pin1, Neurodevelopment, Neurodegeneration, Neuronal apoptosis, Alzheimer’s disease

## Abstract

The peptidyl-prolyl isomerase Pin1 is a unique enzyme catalyzing the isomerization of the peptide bond between phosphorylated serine-proline or threonine-proline motifs in proteins, thereby regulating a wide spectrum of protein functions, including folding, intracellular signaling, transcription, cell cycle progression, and apoptosis. Pin1 has been reported to act as a key molecular switch inducing cell-type-specific effects, critically depending on the different phosphorylation patterns of its targets within different biological contexts. While its implication in proliferating cells, and, in particular, in the field of cancer, has been widely characterized, less is known about Pin1 biological functions in terminally differentiated and post-mitotic neurons. Notably, Pin1 is widely expressed in the central and peripheral nervous system, where it regulates a variety of neuronal processes, including neuronal development, apoptosis, and synaptic activity. However, despite studies reporting the interaction of Pin1 with neuronal substrates or its involvement in specific signaling pathways, a more comprehensive understanding of its biological functions at neuronal level is still lacking. Besides its implication in physiological processes, a growing body of evidence suggests the crucial involvement of Pin1 in aging and age-related and neurodegenerative diseases, including Alzheimer’s disease, Parkinson disease, frontotemporal dementias, Huntington disease, and amyotrophic lateral sclerosis, where it mediates profoundly different effects, ranging from neuroprotective to neurotoxic. Therefore, a more detailed understanding of Pin1 neuronal functions may provide relevant information on the consequences of Pin1 deregulation in age-related and neurodegenerative disorders.

## Background

### The Peptidyl-prolyl Isomerase Pin1

Discovered in 1996 as a protein associating with NIMA (never in mitosis) regulating mitosis [1], the peptidyl-prolyl isomerase Pin1 (protein interacting with NIMA-1) is an ubiquitously expressed *cis/trans* isomerase targeting the phosphorylated serine-proline (pSer-Pro) or threonine-proline (pThr-Pro) motifs [1], belonging to the evolutionarily conserved family of PPIase (peptidyl-prolyl *cis/trans* isomerase). The WW domain on the N-terminus specifically interacts with pSer-Pro or pThr-Pro motifs [2], while the PPIase domain on the C-terminus is responsible for its catalytic activity [3]. Substrate recognition by Pin1 requires phosphorylation of Ser-Pro and Thr-Pro motifs by proline-directed kinase family, including cyclin-dependent kinases (CDKs), mitogen-activated protein kinases (MAPKs), and dual-specificity tyrosine-phosphorylation regulated protein kinase (DYRK). Moreover, Pin1 activity is controlled by protein kinase phosphorylation, as demonstrated by Pin1 phosphorylation in the WW domain, responsible for its increased or decreased binding to the pSer/pThr-Pro motif in substrates [4, 5].

The ensuing conformational changes induced by Pin1 on its protein substrates, as consequence of prolyl-isomerization, produce a variety of functional effects (e.g., substrate stability, catalytic activity, protein-protein interaction, and subcellular localization), thus impinging on several cellular processes, including cell cycle, transcription, and cell fate commitment [6, 7]. In cells, Pin1 has been widely investigated as mitotic regulator, with a fundamental role in checkpoint mechanisms in the cell cycle [8]. However, besides its role in cell cycle progression, Pin1 has been found to interact and regulate also non-nuclear targets with roles in apoptosis, endocytosis, protein translation, maintenance of the cytoskeleton, and neuronal function [6]. Given the role of Pin1 as regulator of cell function by fine-tuning cellular pathways downstream to phosphorylation signaling, perturbation in intracellular pathways and/or deregulation of Pin1 expression/activity, albeit in different directions, has been reported to be implicated in several pathological conditions, such as cancer and neurodegenerative diseases [9].

## Main Text

**Fig. 1:**
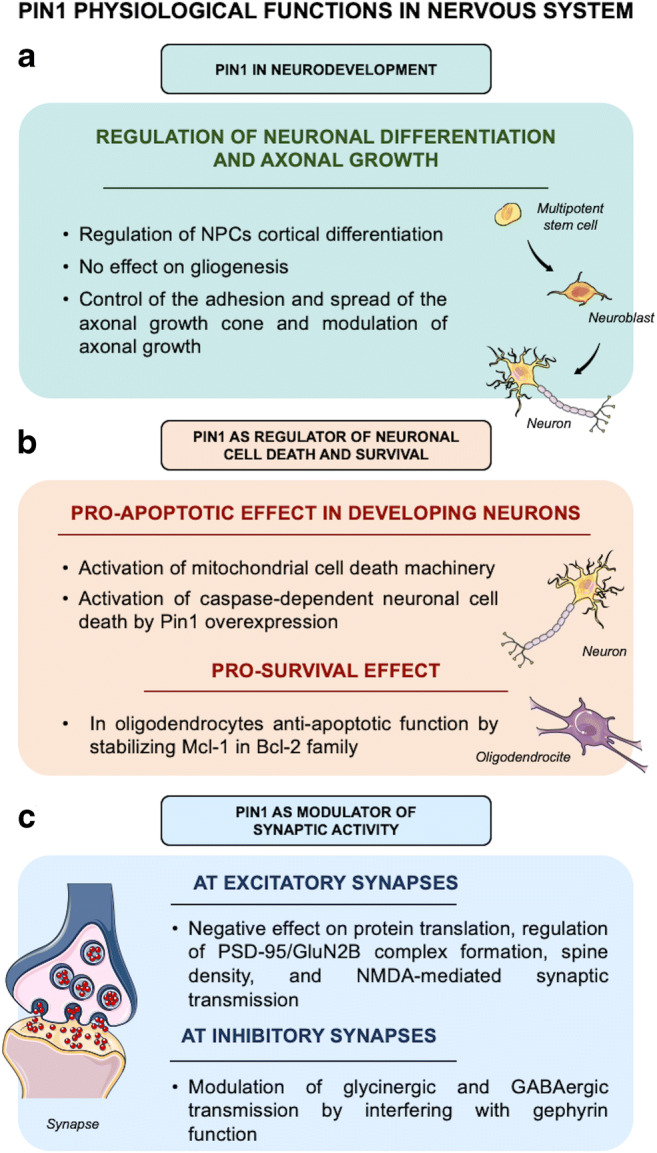
The physiological functions of Pin1 in the nervous system. **a** Pin1 is implicated in neurodevelopment, where it tightly regulates neuronal differentiation and axonal growth. In particular, Pin1 is highly expressed during neurodevelopmental stages, where it controls cortical differentiation of neuron progenitor cells (NPCs), by acting on β-catenin pathway, without affecting gliogenesis. Moreover, Pin1 is required for the development of the central nervous system for axonal growth during embryonic development and for establishing a proper axonal connectivity, by controlling the adhesion and spread of the axonal growth cone. **b** Pin1 acts also as modulator of synaptic activity. At glutamatergic synapses, Pin1 is catalytically present in dendrites, where, under basal conditions, it inhibits protein translation, required for late LTP maintenance, and negatively regulates PSD95/GluN2B complex formation, as well as spine density, and NMDA-mediated synaptic transmission. At glycinergic synapses, Pin1 interacts with gephyrin and alters its overall conformation, thereby affecting the function of glycine receptors. At GABAergic synapses, it inhibits the ability of neuroligin 2 to interact with the scaffolding protein gephyrin. **c** Pin1 is a key context–dependent signal transducer of neuronal cell death and survival signals. In developing neurons, Pin1 binds and stabilizes JNK-phosphorylated forms of BIM_EL_, protecting it from proteasomal degradation and thereby activating the mitochondrial cell death machinery via c-Jun. In addition, Pin1 overexpression overrides NGF-derived survival signals and triggers caspase-dependent neuronal cell death. Besides such pro-apoptotic action, Pin1 also mediates pro-survival effects: as shown by various observation, as reported in the text, and in in vitro experiments on oligodendrocytes, where Pin1 exerts an anti-apoptotic function by binding and stabilizing the anti-apoptotic Bcl-2 family protein Mcl-1, a pro-survival member of the Bcl-2 family, in the cytosol

By controlling the change of the backbones of several cellular substrates, Pin1 acts as key fine-tuner and amplifier of multiple signaling pathways, thereby inducing many functional consequences both in physiological and pathological conditions. In this review, we will critically discuss the highly pleiotropic and context-dependent nature of Pin1 functional activity, which emerges to be strictly related to the phosphorylation patterns of its cellular substrates. In particular, we will specifically focus on Pin1 functions in neurons, starting from its implication in neurodevelopment to its role in cellular homeostasis in adult neurons (Fig. 1). Moreover, we will discuss evidence from the literature supporting the notion of a differential role of Pin1 within the different neurodegenerative diseases (Table 1).

**Table 1:** Summary of Pin1 changes in neurodegenerative diseases

Neurodegenerative disease	Pin 1 changes observed in human samples	Pin 1 putative role	References
Alzheimer’s disease	Pin1 has been reported to be oxidatively modified, with consequent reduced activity and expression in hippocampus from MCI and AD patients compared to age-matched controls.	Protective role against age-associated neurodegeneration	[10, 11]
	Change in Pin1 neuronal localization, with a shift from nucleus to cytoplasm, have been found in p*ostmortem* human brains from AD patients compared to age-matched controls.		[12–14]
	Loss of Pin1 within the synapses of human frontal tissues from AD patients compared to age-matched control brains.		[12]
	Pin1 co-localized with deposits of aggregated tau.		[15]
Frontotemporal dementias	Overall reduction of Pin1 and redirection of the predominantly nuclear Pin1 into the neuronal cytoplasm in FTD *postmortem* brains compared to normal brains.	Protective role against age-associated neurodegeneration	[16]
	A trend for downregulation of Pin1 in human non-motor cortex and in the spinal cord derived from patients with FTLD-U.		[17]
Parkinson disease	Pin1 has been reported to accumulate in the Lewy Bodies of human PD brains and to co-localize with α-synuclein inclusions.	Neurotoxic action by promoting with α-synuclein inclusions	[18]
	Increased levels of Pin1 have been observed in pigmented dopaminergic neurons in p*ostmortem* human brains from PD patients.	Neurotoxic action contributing to dopaminergic neurodegeneration	[19]
Huntington disease	No data on human samples are available, but only in vitro and in vivo preclinical results.	Neurotoxic action by promoting p53-dependent neuronal apoptosis induced by mutant huntingtin	[20]
Amyotrophic lateral sclerosis	Downregulation of Pin1 expression in the spinal cord and non-motor cortex of a cohort of ALS patients.	Protective role against neurofilament-H hyperphosphorylation and its perikaryal accumulation in in vitro models	[17, 21]

## Pin1 in Neurons

Pin1 is widely expressed in human tissues including the central and peripheral nervous system [22]. In particular, it is highly expressed in terminally differentiated and post-mitotic neurons, specifically enriched at mitochondrial membranes [23], but also present in neuronal cytosol, dendrites [24, 25], and distal axons [26]. Consistently with its neuronal localization, Pin1 has been reported to regulate a variety of neuronal processes, such as neurodevelopment, neuronal differentiation [27], dendritic protein synthesis [25], and axonal growth and guidance [26] (Fig. 1). However, while the role of Pin1 in proliferating cells and, in particular, in the field of cancer, has been widely characterized, less is known about the functions of Pin1 during the development of the nervous system and in post-mitotic adult neurons. In fact, despite studies reporting the effects of Pin1 on its neuronal substrates or specific intracellular signaling pathways, as detailed below, a more comprehensive understanding of its biological role at neuronal level is still lacking.

### The Physiological Role of Pin1 in Neurodevelopment

Pin1 has been found highly expressed during neurodevelopmental stages, playing a key role in regulating cortical differentiation of neuron progenitor cells (NPCs). Pin1 knockdown in mice has been observed to reduce NPC differentiation, while Pin1 overexpression has been reported to enhance it, without affecting gliogenesis, thus suggesting that Pin1 may specifically promote neuronal but not glial differentiation of NPCs [27]. In accordance with the pattern of Pin1 expression in developing brains, Pin1 knockdown inhibited NPC differentiation into migrating immature neurons at E15.5, without affecting NPCs expansion phase [27]. In addition, Pin1 knockdown specifically inhibited the birth of upper layer neurons, but not that of the lower layers in the cerebral cortex. Such reduction in the upper layer neurons, induced by Pin1 knockdown, was also confirmed in mice motor cortex at late embryonic stages and in the neonatal stage, where mice displayed a severe impairment in neonatal motor activity [27]. The molecular mechanism by which Pin1 has been reported to regulate NPC differentiation seems to rely on its interplay with β-catenin, identified as Pin1 major substrate in NPCs by proteomic approach [27]. Pin has been demonstrated to bind and stabilize β-catenin conformation at late stage during mouse brain development [27]. Consistently, Pin1 knockout reduced β-catenin in NPCs at late stages during brain development [27].

Moreover, conformational changes induced by Pin1 represent an important regulatory mechanism also in axonal growth during embryonic development [26]. Pin1 has been shown to bind and stabilize CDK5-phosphorylated CRMP2A (collapsin response mediator protein 2 A) [26]. The kinase-mediated phosphorylation of CRMP2 reduces its affinity to tubulin, thus promoting microtubule disassembly. Notably, Pin1 knockout, knockdown, and inhibition reduced CRMP2A levels, specifically in distal axons, and inhibited axon growth, fully rescued by Pin1 overexpression [26]. Noteworthy, in Pin1 knockout mouse embryos, defects in developmental axon growth both in the peripheral and central nervous system have been observed at E12.5, with cranial and spinal nerves displaying stunted and less branched neurite processes [26]. Moreover, entorhinal hippocampal perforant projections were significantly shorter in Pin1 knockout embryos at E15.5 compared to wild-type mice [26]. Interestingly, in newborn and adult Pin1 knockout mice, the entorhino-hippocampal projections were detected in the stratum lacunosum-moleculare as in Pin1 wild-type mice, thus indicating that the previously mentioned defects in Pin1 knockout embryos were later corrected [26].

Among the functions of Pin1 in the developing brain, Pin1 has been also recently implicated in the regulation of the axonal growth cone motility [28]. In particular, in embryonic rat brain, Pin1 has been found to directly interact and regulate the dephosphorylation of the myristoylated alanine-rich C kinase substrate (MARCKS), a protein enriched in the developing brain, modulating neuronal spreading and migration, and stabilizing the adhesion complex at the growth cone [28]. Pin1 seems to be required for the normal development of the central nervous system for establishing a proper axonal connectivity, by controlling adhesion and spread of the axonal growth cone. Accordingly, in Pin1 knockout mice, Sosa et al. described the presence of morphological alterations in the corpus callosum and cerebral cortex fibers [28]. In particular, a thinner corpus callosum in the midline, along with a reduction in the amount of fibers crossing over, has been observed in Pin1 null mice compared to control mice, indicating a defective connection between the two cortical hemispheres [28].

Taken together, such evidence indicating the correlation between Pin1 deregulation and morphological and functional modifications during brain development strongly points the critical involvement of Pin1 in embryogenesis and neurodevelopment.

### Pin1 as Regulator of Neuronal Apoptosis

Data from literature suggest that, in the nervous system, the regulation of neuronal cell death and survival by the prolyl isomerase Pin1 critically depends on the tissue context and the developmental stage, with Pin1 capable to trigger both pro-survival [29] and pro-apoptotic pathways [24, 30]. Accordingly, Pin1 has been shown to promote cell survival by maintaining the normal mitochondrial homeostasis. Consistently, Pin1 has been reported to exert an anti-apoptotic function in adult mouse oligodendrocytes by binding and stabilizing the anti-apoptotic protein Mcl-1 (myeloid cell leukemia sequence-1), a pro-survival member of the Bcl-2 family, in the cytosol [29]. In a mouse model of spinal cord injury, obtained by hemisection at thoracic level, JNK3 (c-Jun N-terminal kinase-3) has been demonstrated to perturb the interaction between Pin1 and Mcl-1 by phosphorylating the latter at Ser121, thereby inducing proteasome-mediated degradation of Mcl-1 and, ultimately, leading to cytochrome c release from mitochondria [29], a fundamental step to activate caspase pathways triggering apoptotic process. According to Pin1 anti-apoptotic function, in Pin1^−/−^ mice, Mcl-1 levels were reduced, cytochrome c release is constitutive in the absence of injury, and apoptosis significantly increased after injury [29]. Noteworthy, given the relevance of mitochondrial dysfunction in the pathophysiology of several neurodegenerative diseases, further investigations are required to investigate the JNK3-mediated perturbation of Pin1/Mcl-1 interaction, necessary for maintaining mitochondrial homeostasis, also in the central nervous system.

In contrast with these findings reporting Pin1 pro-survival effects in the nervous system, Pin1 has been also demonstrated to act as positive regulator of programmed cell death specifically in developing neurons [24, 30]. Consistently, Becker and Bonni provided evidence showing Pin1 implication in the regulation of neuronal apoptosis [24]. In post-natal cerebellar granule neurons, Pin1 has been shown to mediate the activation of the mitochondrial cell death machinery following trophic factor deprivation [24]. In particular, Pin1 has been reported to specifically bind and stabilize JNK-phosphorylated at Ser65 forms of BIM_EL_ (Bcl-2-interacting mediator of cell death) via its WW domain, protecting BIM_EL_ from proteasomal degradation and thereby activating the mitochondrial cell death machinery via c-Jun [24]. Notably, a significant proportion of Pin1 in neural cells is tethered to the mitochondrial membrane, where it engages in a physical complex with the JNK scaffold protein JIP3. Later, Barone et al. confirmed that, in primary cultures of sympathetic neurons from superior cervical ganglia of newborn (post-natal day 0–1) mice, the overexpression of catalytically active Pin1 was capable to override NGF (nerve growth factor)–derived survival signals and to trigger caspase-dependent cell death of neurons, which was accompanied by the accumulation of Ser63-phosphorylated c-Jun in neuronal nuclei [30]. In contrast, the downregulation of Pin1 expression suppressed the accumulation of phosphorylated c-Jun, as well as the consequent release of cytochrome *c* from mitochondria and delayed cell death [30]. Moreover, ectopic Pin1-induced cell death was prevented by the expression of dominant-negative c-Jun [30].

Overall, these findings suggest that Pin1 may participate to the regulation of neuronal cell death specifically in developing neurons, but it promotes neuronal survival in adult neurons. However, future studies are required to define Pin1 role as pro-apoptotic and/or pro-survival regulator within neurons at different stages of neuronal development and to investigate its potential interaction with other apoptotic and metabolic regulators residing at mitochondria. In addition, as widely reported in cancer cell lines [31], Pin1 intimately interacts with the tumor suppressor protein p53 sculpting the active pro-apoptotic shape of p53, thus promoting its activity as inducer of cellular death [32]. However, data concerning Pin1/p53 interplay in neuronal context and its implication in the regulation of cell fate are still lacking. In this regard, although several cellular components of the cell death machinery are shared by both post-mitotic neurons and proliferating cells, their functions in apoptotic processes can be profoundly different. As an example, while the phosphorylation of BIM_EL_, induced by specific stimuli, triggers neuronal cell death, the same event in non-neuronal cells promotes survival [23].

### Pin1 as Modulator of Synaptic Activity

By performing immune-electron microscopy, Westmark and collaborators demonstrated that Pin1 is highly expressed and catalytically active in dendritic shafts and spines in rodent cortex and hippocampus, with a preferential post-synaptic localization, where, under basal conditions, it inhibits protein translation [25]. In particular, Pin1 has been reported to be associated with Shank proteins at dendritic rafts and with post-synaptic density protein-95 (PSD-95), indicating its potential involvement both in regulating signal transduction at dendritic drafts and signal processing at the PSD [12, 33]. Notably, at excitatory synapses, Pin1 has been described to negatively regulate PSD-95/GluN2B complex formation, as well as spine density, and NMDA (N-methyl D-aspartate)–mediated synaptic transmission [33]. In parallel, Pin1 has been also reported to be involved in the regulation of inhibitory transmission, by modulating neuroligin 2 (NL2)/gephyrin interaction at inhibitory GABAergic synapses [34, 35]. Therefore, an emerging role of Pin1 as modulator of synaptic activity has been proposed. However, despite sparse information about Pin1 involvement in synaptic activity, as discussed in the following sections, the balance of Pin1 effects on excitatory and inhibitory transmission, under basal conditions, remains to be unveiled.

### Pin1 in Excitatory Transmission

Westmark et al. demonstrated that Pin1 is present and catalytically active at dendrites of glutamatergic synapses, where it inhibits protein synthesis under basal conditions [25]. Notably, protein synthesis is essential for the formation of long-term memory and the maintenance of long-term forms of synaptic plasticity, such as late LTP (long-term potentiation). Interestingly, while basal synaptic transmission, as measured by the field excitatory post-synaptic potential (fEPSP) slope versus voltage, did not differ in hippocampal slices from Pin^−/−^ mice compared to wild-type controls, paired-pulse facilitation, a form of short-term synaptic plasticity, was increased in Pin^−/−^ mice, thus suggesting that Pin1 may affect neurotransmitter release [25]. Furthermore, during a protocol designed to stimulate late LTP (four high-frequency trains of stimuli), hippocampal slices from Pin^−/−^ mice displayed normal early LTP, but significantly enhanced protein synthesis–dependent late LTP, compared to wild-type slices [25]. Such increase was prevented by protein synthesis inhibitors [25].

Moreover, at post-synaptic terminal, Pin1 has been demonstrated to directly interact with PSD-95, a membrane-associated guanylate kinase acting as scaffold protein at excitatory post-synaptic densities and anchoring NMDA receptor via GluN2-type receptor subunit [33]. In particular, Pin1 has been reported to interact with PSD-95 at specific Ser/Thr-Pro consensus motifs localized in the linker region connecting PDZ2 and PDZ3 domains [33]. Upon binding, Pin1 induces structural modifications in PSD-95, thereby inhibiting its ability to interact with NMDA receptors. Notably, electrophysiological experiments showed that, in hippocampal slices from Pin1^−/−^ mice, larger NMDA-mediated synaptic currents, evoked in CA1 principal cells by Schaffer collateral stimulation, were detected [33]. Such effect was also observed in cultured hippocampal cells expressing a PSD-95 mutant, unable to undergo prolyl-isomerization, further corroborating the hypothesis that Pin1 isomerase activity on PSD-95 is pivotal. Moreover, a significant increase in spine density, due to a selective gain in mushroom spines, was observed in Pin1^−/−^ pyramidal neurons [33].

Overall, these data suggest that, under basal conditions, Pin1 negatively regulates the induction of dendritic translation, required for late LTP maintenance, as well as PSD-95/GluN2B complex formation, spine density, and NMDA-mediated synaptic transmission at excitatory synapses [25, 33]. However, despite such sparse information concerning Pin1 interplay with excitatory transmission, a comprehensive understanding of Pin1 synaptic effects has still to be unveiled. In this regard, behavioral tests in germ-line *Pin1* knockout mice would be useful to assess whether potential Pin1-related changes not only in LTP but also in LTD (long-term depression) are accompanied by modifications in spatial memory, contextual fear memory, and social behavior.

Noteworthy, Tang et al. recently demonstrated that Pin1 directly interacts with NR2A- and NR2B-containing NMDA receptors, but not AMPA (α-amino-3-hydroxy-5-methyl-4-isoxazole propionic acid) receptors in the hippocampus of epileptic mouse models and suggested the implication of Pin1/NMDA receptors complex in epileptic seizures [36]. Notably, Tang et al. reported a reduction in Pin1 protein levels in the neocortex of patients with temporal lobe epilepsy compared to controls, a decrease observed also in the hippocampus and cortex of chronic pilocarpine epileptic mouse model [36]. These findings suggest that epileptic seizures may downregulate Pin1 expression. However, further studies are needed to clarify whether dysregulation of such Pin1-based mechanism may participate to epileptogenesis.

### Pin1 in Inhibitory Transmission

At inhibitory synapses, Pin1 was found to interact with gephyrin, the functional homolog of PSD-95, and to alter its overall conformation, thereby affecting the function of glycine receptors [34]. Later, Antonelli et al. showed a mechanism by which Pin1 may affect the efficacy of GABAergic transmission by modulating NL2/gephyrin interaction at inhibitory GABAergic synapses [35]. In particular, NL2 has been reported to undergo proline-directed phosphorylation at Ser714-Pro consensus site and, subsequently, Pin1-mediated *cis/trans* isomerization [35]. Such post-phosphorylation prolyl-isomerization by Pin1 has been found to inhibit the ability of NL2, a cell adhesion molecule of the neuroligin family, enriched at GABAergic synapses, to interact with the scaffolding protein gephyrin [35]. Accordingly, immunocytochemical analysis demonstrated that NL2/gephyrin complexes were enriched at GABAergic post-synaptic sites in the hippocampus of Pin1-knockout mice (Pin1^−/−^) [35]. This enrichment was accompanied by an enhanced synaptic recruitment of GABA_A_ receptors and by a concomitant increase in the amplitude, but not in frequency, of spontaneous GABA_A_-mediated post-synaptic currents. Thus, Pin1-mediated modulation of NL2/gephyrin interaction represents a novel mechanism by which Pin1 may impinge on GABAergic transmission, thus possibly playing a key role in remodeling GABAergic synapses.

## Pin1 in Aging and Neurodegenerative Diseases

Data from the literature indicates that Pin1 plays a central role in regulating aging process in vivo. Accordingly, Pin1-knockout mice are viable and, despite transitory changes observed in the neurodevelopmental studies reported above and that are later corrected, they develop with a normal phenotype for an extended period of time [37]. However, adult Pin1-deficient mice display a range of abnormalities, including reduced body size, changes in skeletal or muscular structure (e.g., osteoporosis, lordokyphosis), retinal degeneration, and widespread signs of premature aging and neurodegeneration, such as acceleration of telomere shortening, massive tau phosphorylation and deposition in typical paired helical filaments, increased production of β-amyloid 42 (Aβ_42_), loss of motor coordination and behavioral defects, neuronal loss, and degeneration [38]. Hence, ablation of Pin1 gene results in a phenotype that recapitulates the phenomena associated with aging and some neurodegenerative conditions, in the absence of defective transgenes such as mutant human APP (amyloid precursor protein) or tau [39]. Accordingly, a growing body of evidence suggests that Pin1 plays a crucial role in the pathophysiology of several neurodegenerative diseases. In the context of Alzheimer’s disease (AD), the *cis/trans* isomerase Pin1 has been proposed to protect against age-dependent neurodegeneration, by directly restoring the conformation and function of phosphorylated tau [13], as well as by promoting the non-amyloidogenic processing of APP and, consequently, reducing Aβ production [40]. Notably, in AD brains, changes in Pin1 neuronal localization, with a shift from nucleus to cytoplasm, have been observed in *postmortem* human brains, with a significant overall reduction of Pin1 compared to age-matched controls [13]. Such redirection of Pin1 has been also reported in brains of patients with frontotemporal dementias. In contrast with Pin1 neuroprotective role in AD, Pin1 has been found to accumulate in the Lewy bodies of human PD (Parkinson disease) brains and to contribute to the formation of α-synuclein inclusions [18]. Moreover, increased levels of Pin1 have been reported in pigmented dopaminergic neurons in PD human brains, where it mediated a neurotoxic action contributing to dopaminergic neurodegeneration [19]. Furthermore, in Huntington disease (HD), Pin1 has been found to promote p53-dependent neuronal apoptosis, induced by mutant huntingtin [20]. In addition, inhibition of Pin1 has been shown to reduce neurofilament (NFT)-H hyperphosphorylation and its pathological perikaryal accumulation in in vitro models of amyotrophic lateral sclerosis (ALS) [21]. In contrast, in mice intracerebrally infected with RLM (Rocky Mountain Laboratory) prion strain—a mouse-adapted scrapie prions resembling the pathological features occurring in prion protein diseases—neither total depletion nor reduced levels of Pin1 have been found to affect the process of prion protein misfolding or to alter the typical clinical and neuropathological features of the disease both in hemizygous Pin1^±^ and knockout Pin1^−/−^ mice [41]. Therefore, a differential role of Pin1 within the different neurodegenerative diseases clearly emerges (Table 1) and it is still subject of scientific debate. Such diverse implication of Pin1 in neurodegeneration may rely, at least in part, on the different phosphorylation patterns of Pin1 targets in the different cellular and pathological context.

### Pin1 in Alzheimer’s Disease

The first evidence of Pin1 involvement in neurodegenerative disorders, such as AD, dates back to 1999, when elevated levels of Pin1 binding to NFT-rich cytoplasm of AD neurons were reported [13]. Later, Pin1 has been reported to be oxidatively modified, with consequent reduced activity and expression in hippocampus from MCI (mild cognitive impairment) and AD patients compared to age-matched controls [10, 11]. Moreover, using light microscopy, change in Pin1 intracellular localization—predominantly nuclear—has been also observed in neurons from AD patients, where Pin1 was localized to neuronal cytoplasm and perikaryal NFTs [13]. Notably, after the application of exogenous recombinant Pin1 to AD brain sections, it has been observed that recombinant Pin1 was bound to the phosphorylated Thr231 residue of tau and it was sequestered within tangles, thereby reducing the amount of soluble Pin1 protein [13]. In addition, Pin1 activity has been reported to directly restore the conformation and function of phosphorylated tau by indirectly promoting its dephosphorylation [13]. In particular, Pin1 has been found to bind to tau at phosphorylated Thr231-Pro, thereby stimulating PP2A-driven dephosphorylation and restoring its microtubule-binding functions [13, 42]. However, the hypothesis that aggregated tau sequesters and depletes soluble Pin1 reserve is controversial. In fact, Dakson and collaborators, by analyzing the content of Pin1 in hippocampal and cortical neurons of brains from AD patients, demonstrated an increase in Pin1 immunoreactive granules within the hippocampal regions of CA2, CA1, subiculum, and presubiculum, whereas minimal occurrence or complete absence have been reported in cortical areas with prominent NFT pathology, such as the entorhinal and temporal cortices [43]. Thus, the incidence of Pin1 immunoreactive granules seems not to correlate with the frequency and distribution of NFT pathology, as well as with the presence or absence of Aβ [43]. In young brains, absent or mild Pin1 immunoreactivity has been observed [43].

Besides Pin1 correlation to tau, Pastorino et al. demonstrated that Pin1 also regulates APP processing and Aβ production [40], by binding to the phosphorylated Thr668-Pro motif of APP and accelerating its intracellular domain isomerization [40]. In particular, they reported that the *cis* phosphorylated Thr668-Pro conformation promoted the amyloidogenic processing of APP, whereas the *trans* conformation the non-amyloidogenic pathway. By catalyzing such conversion, Pin1 has been demonstrated to promote the non-amyloidogenic processing of APP [40]. In mice, Pin1 knockout, alone or in combination with overexpression of mutant APP, has been linked to increased amyloidogenic APP processing, with a selective enhancement in insoluble Aβ_42_ levels in an age-dependent manner [40]. These data are intriguing since they suggest that the Pin1 mutation is sufficient alone to induce an age-dependent brain amyloidosis. In particular, while, in Pin^−/−^ mice at 2–6 months of age, no change in the levels of Aβ_42_ was detected, at 15 months, a significant increase in insoluble Aβ_42_ content was observed compared to Pin^+/+^ mice [40]. Such increase in insoluble Aβ_42_ levels was accelerated by APP overexpression in Pin^−/−^ transgenic mice (*Tg2576*), where enhanced insoluble Aβ_42_ by 46% was detected at 6 months compared to Pin^+/+^ littermates [40].

Recently, Xu et al. demonstrated a pathological loss of Pin1 within the synapses of human frontal tissues from AD patients compared to age-matched control brains [12]. In particular, total synaptic Pin1 protein content was significantly reduced by 39% in human AD patient frontal cortical tissues compared to controls [12]. In C57/BL6 cortical neurons, the pharmacological inhibition of Pin1 catalytic activity with PiB (diethyl-1,3,6,8-tetrahydro-1,3,6,8-tetraoxobenzol-phenanthroline-2,7-diacetate) or Pin1 siRNA-mediated knockout induced an increase in ubiquitin-regulated modification of PSD proteins and a reduction in Shank3 protein levels [12], an observation consistent with Shank3 protein loss and enhanced ubiquitination described in AD brains [44, 45]. Such effects induced by Pin1 loss may possibly contribute to pathological changes in PSD structures and synaptic damage [12]. Based on evidence reporting a reduced activity of Pin1 in the early stage of the disease, as observed in MCI, loss of Pin1 may represent an early event participating to the pathological alterations of synaptic proteins and, ultimately, leading to synaptic loss or alternatively the consequence of a reduced number of synapses. Notably, since Pin1 has been found downregulated and oxidatively modified in MCI patients [10, 11], it might be further investigated as potential biomarker to detect neurodegenerative processes occurring early in the progression of AD [46].

The emerging picture is that of a neuroprotective role for Pin, the loss of which, observed both in MCI and AD brains, may accelerate both neurofibrillary tangles and senile plaques formation and impair synaptic homeostasis. Consistently, a functional polymorphism, rs2287839, in Pin1 promoter has been reported to associate with a 3-year delay in the average age-at-onset of late-onset AD in a Chinese population [47]. Specifically, this polymorphism, located within the consensus motif for the brain-selective transcription factor AP4, almost completely prevented AP4 binding to Pin1 promoter and, consequently, Pin1 expression was unresponsive to the repressive effect of AP4 [47]. In contrast, other polymorphisms in the promoter region of *PIN1* gene have been related to an increased risk of AD. As an example, a study by Segat et al. identified two single nucleotide polymorphisms at positions − 842 and − 667 in the promoter region of *PIN1* gene and reported a significantly higher percentage of − 842C allele carriers in AD subjects compared to controls, suggesting that the inheritance of such allele may alter Pin1 expression and, consequently, enhance the risk of developing AD [48].

### Pin1 in Frontotemporal Dementia

As observed in AD brains, tau hyperphosphorylation in the NFTs is accompanied by the redirection of the predominantly nuclear Pin1 into the neuronal cytoplasm, as well as by Pin1 deficits throughout subcellular compartments. Intriguingly, a similar redistribution and reduction of Pin1 have been observed in a range of frontotemporal dementias (FTDs), both with tau pathology (FTD with tau mutation, Pick disease, corticobasal degeneration) and without tau pathology (frontotemporal lobar degeneration with motor neuron-type inclusions, and neuronal intermediate filament inclusion disease) [16]. Accordingly, in neurons derived from the middle frontal gyrus of control and FTD *postmortem* brains, Thorpe and collaborators found a redistribution of Pin1 from the nucleus to the cytoplasm in all the FTD cases, compared to normal brains, which conversely displayed a prevalent nuclear localization of Pin1 [16]. This observed redirection of the mitotic regulator Pin1 from neuronal nucleus to cytoplasm is likely to depend on the presence of p-tau, as well as on the increased amount of its other target phosphoproteins in neuronal cytoplasm, such as mitotic phosphoepitopes and cell cycle–related proteins [49–52]. Accumulation of these proteins has been observed in different pathological contexts (e.g., AD, FTDP-17, progressive supranuclear palsy, corticobasal degeneration) [53] and described as manifestations of interrupted mitotic process leading to cytoskeletal abnormalities and neuronal apoptosis [16]. However, further investigations are necessary to evaluate whether Pin1 redirection to the cytoplasm represents an early event occurring and mediating the neurodegenerative processes or the result of it.

Notably, Iridoy et al. recently analyzed the pattern of Pin1 expression by using a proteomic approach, demonstrating a trend for downregulation of Pin1 in human non-motor cortex and in the spinal cord derived from patients with ubiquitin frontotemporal lobar degeneration (FTLD-U), the most common form of FTD [17].

### Pin1 in Parkinson Disease

The prolyl isomerase Pin1 has been also implicated in the pathogenesis of PD. Ryo and collaborators demonstrated that Pin1 accumulated in the Lewy bodies of human PD brains and co-localized with α-synuclein inclusions [18]. In particular, Pin1 overexpression has been observed to facilitate the formation of α-synuclein inclusions in 293T cells transfected with α-synuclein, while dominant-negative Pin1 abrogated it [18]. Specifically, Pin1 overexpression has been reported to enhance the half-life and insolubility of α-synuclein, as well as to bind to synphilin-1, an α-synuclein partner, thereby promoting its interaction with α-synuclein and the formation of α-synuclein cytoplasmic inclusions [18]. Later, Ghosh et al. provided evidence regarding the upregulation of Pin1 due to neurotoxic stress and its role as pro-apoptotic factor contributing to dopaminergic neuronal degeneration [19]. Indeed, Pin1 has been reported to be significantly upregulated in *postmortem* human midbrain of PD patients in comparison with aged-matched controls [19], as well as in vitro in dopaminergic MN9D neurons, treated with 1-methyl-4-phenylpyridinium (MPP^+^), and in the substantia nigra of the 1-methyl-4-phenyl-1,2,3,6-tetra-hydropyridine (MPTP)–induced PD mouse model [19]. Notably, siRNA-mediated knockdown of Pin1 has been observed to prevent MPP^+^-induced caspase-3 activation and DNA fragmentation, thus suggesting that Pin1 may induce apoptosis in dopaminergic neurons [19]. Accordingly, different pharmacological Pin1 inhibitors, such as juglone, reduced MPP^+^-driven Pin1 upregulation, α-synuclein aggregation, caspase-3 activation, and neuronal death [19]. Furthermore, juglone treatment in the MPTP mouse model of PD suppressed Pin1 levels and ameliorated functional locomotor deficits, dopamine depletion, and nigral dopaminergic neuronal loss [19]. Noteworthy, the Pin1 inhibitor PiB reduced α-synuclein protein aggregation, induced by MPP^+^, in the N27 dopaminergic cell models, thereby indicating that upregulation of Pin1, driven by neurotoxic pulse, might contribute to α-synuclein protein misfolding and aggregation [19]. However, the precise intracellular mechanism of Pin1 upregulation in α-synuclein misfolding and aggregation has to be unveiled.

Taken together, such results provide evidence of a potential pro-apoptotic role of Pin in dopaminergic neurons, indicating that its upregulation may represent a critical neurotoxic event in the pathogenesis of PD.

### Pin1 in Huntington Disease

HD is a dominantly inherited neurodegenerative disorder, caused by CAG repeat expansion in the gene codifying for huntingtin protein and characterized by massive loss of medium spiny neurons in the striatum [54]. Among the different mechanisms by which mutated huntingtin triggers striatal neurodegeneration, DNA damage and neuronal apoptosis have been proposed as key mechanisms. In this regard, the tumor suppressor p53 has been found to mediate toxic effects of mutated huntingtin with expanded polyglutamine [20, 55]. Mutated huntingtin has been reported to bind to p53 and to increase p53 levels in whole tissue lysates of *postmortem* cerebral cortex and striatum of HD patients, as well as to induce its transcriptional activity [55]. Later, Grison et al. demonstrated that, in *postmortem* brains of HD patients, the expression of mutated huntingtin evoked a canonical DNA damage response and was correlated to an enhanced phosphorylation of p53 at Ser46 [20]. Such phosphorylation generated a target site for Pin1, thereby promoting p53 interaction with Pin1 and the dissociation of p53 from the apoptosis inhibitor iASPP in in vitro models, thereby inducing the expression of its apoptotic target genes [20]. Noteworthy, Ser46 phosphorylation, triggered by severe or persistent stress, has been reported to be the major event in shifting p53 response from cell cycle arrest to apoptosis and the isomerization by Pin1 as a key step to stimulate the apoptotic potential of p53. Furthermore, a toxic feedback loop has been demonstrated, where mutated huntingtin promotes Pin1-mediated activation of p53 that, in turn, induces the expression of mutated huntingtin [56], Therefore, such results provide evidence of a potential mechanism through which Pin1/p53 pathways participate to the induction of neuronal apoptosis in response to mutated huntingtin.

### Pin1 in Amyotrophic Lateral Sclerosis

ALS is a neurodegenerative disorder that affects the upper and lower motor neurons, leading to paralysis of voluntary muscles, dysphagia, dysarthria, and respiratory failure [57]. Recently, by using a proteomic approach, Iridoy and collaborators demonstrated a significant downregulation of Pin1 expression in the spinal cord and non-motor cortex of a small cohort of patients with ALS [17], indicating Pin1 expression as a potential marker of neurodegeneration. However, the current knowledge about the expression profile of Pin1 in ALS is extremely limited, as well as its involvement in the pathophysiology of this disease. Evidence from the literature suggests that it may promote the abnormal accumulations of phosphorylated neurofilament proteins in the perikaryon, a major hallmark of ALS, as well as of other neurodegenerative diseases [21, 58]. Accordingly, Pin1 has been reported to associate with phosphorylated NF-H in neurons and to co-localize in ALS-affected spinal cord neuronal inclusions [21]. In rat dorsal root ganglion cultures subjected to excitotoxic stress to evoke the accumulation of phosphorylated NF-H within the cell body in order to mimic neurodegeneration, glutamate-induced toxicity has been demonstrated to increase phosphorylated NF-H in perikaryal accumulations that co-localized with Pin1 and induced neuronal apoptosis [21]. Such effects were reduced by pharmacological inhibition or siRNA-mediated downregulation of Pin1 [21], thus suggesting that, upon neurotoxic pulse, Pin1 may promote cell death by stimulating the perikaryal aggregation of phosphorylated NF-H.

## Conclusions

### Pin1 as a Crucial Signal Transducer Acting in a Context-Dependent Manner

By inducing the isomerization of the *cis/trans* configuration of its cellular substrates, Pin1 acts as key fine-tuner and amplifier of multiple signaling pathways, thereby displaying a variety of functional consequences both in physiological and pathological conditions. As discussed in this review, a highly pleiotropic and context-dependent nature of Pin1 functional activity, strictly dependent on the phosphorylation patterns of its cellular targets, clearly emerges. Noteworthy, in the nervous system, Pin1 is fundamental both for embryonic development and cellular homeostasis in adult neurons, due to its role as regulator of cell death and survival. In particular, in developing neurons, it seems to participate to the induction of neuronal cell death, whereas in adult neurons, to promote neuronal survival. Notably, while accumulating evidence has characterized Pin1 role in the regulation of cell fates in cancer, Pin1 functional activity on neuronal homeostasis and, specifically, Pin1 role as pro-apoptotic and/or pro-survival regulator within neurons, at different stages of neuronal development, has still to be unveiled. Moreover, despite sparse evidence supporting Pin1 regulation of protein translation at dendrites and its interaction with specific synaptic substrates at excitatory and inhibitory synapses, the overall balance of its activity at synapses, under physiological conditions, is unknown. However, a comprehensive understanding of Pin1 physiological functions in neurons and, specifically, at synapses is critical to define whether and how an imbalance in of Pin1 activity and/or expression may impact on neuronal homeostasis and, ultimately, contribute to pathological mechanisms. In this regard, our knowledge of Pin1 physiological activity in neurons is extremely limited and some inconsistencies complicate the scenario. As an example, multiple lines of evidence report that in human AD brains, Pin1 activity and protein content are markedly reduced [10, 11] and that in Tg2576 mice, germ-line *Pin1* knockout significantly accelerates AD pathology, as discussed above [15]. However, data from literature paradoxically demonstrate that hippocampal slices derived from germ-line *Pin1* knockout mice showed enhanced, rather than decreased, LTP [25], as well as increased, rather than reduced, hippocampal spine density [33]. Notably, these phenotypes are opposite to those expected from the pathology commonly observed in human AD brains and murine AD models. In this regard, it is tempting to speculate that such discrepancies between spine density and LTP in germ-line *Pin1* knockout and AD mouse models depend upon the model used, one based on the lifelong lack of Pin1 versus the progressive loss of it over time. Accordingly, while germ-line knockout mice harboring a null allele provide appropriate genetic models of inherited disease, conditional gene inactivation seems to be a more appropriate approach to assess the post-development effects of Pin1 loss in adult organisms and to achieve gene inactivation in selected cell types [59]. Hence, depending on the model used, it is possible to differentiate distinct functions of Pin1 in dendritic spine development and spine maintenance. As proof of concept, Stalling et al. recently demonstrated that in Pin1 floxed mice and derived neuronal cultures, post-natal Pin1 loss induced a significant decrease in spine density, rescued by the application of exogenous Pin1, thus suggesting that Pin1 is required for dendritic spine maintenance in mature neurons [60]. Therefore, further investigations should consider this aspect to select more suitable models to assess Pin1 role in age-related pathologies, where Pin1 loss occurs late in the lifespan.

In conclusion, while under physiological conditions, Pin1 activity ensures a homeostatic equilibrium by fine-tuning the ability of cells to transduce a variety of stimuli and to elicit integrated biological responses, in pathological contexts, an imbalance in Pin1 activity and/or expression may exacerbate diseases by hijacking cellular processes regulated by Pin1 to sustain pathological mechanisms [61]. In line with this hypothesis, Pin1 has been reported to play a key role in acute neurological conditions associated with subsequent neurodegeneration, such as ischemic stroke [62], where it promotes neuronal death by acting on Notch1 signaling pathway [62]. Pin1-deficiency has been found to prevent stroke-induced brain damage and neurological deficits in Pin1^−/−^ mice [62]. Such evidence supports the notion that Pin1 is a key molecular switch regulating neuronal cell fates also in pathological conditions. Indeed, profoundly different roles of Pin1, ranging from neuroprotective to neurotoxic, have been observed within different pathological contexts (Table 1), further indicating the context-dependent nature of Pin1 functional activity.

Therefore, Pin1 clearly emerges as a crucial signal transducer that, under normal conditions, regulates the activation of multiple signaling pathways, thereby inducing biological outcomes downstream to a plethora of stimuli, but, when imbalanced, may participate to pathological mechanisms, thus providing a promising therapeutic target in a wide array of pathological conditions. Its chameleonic role increases the difficulties associated with drug interventions targeting it in absence of biomarkers allowing to decide whether a certain status will benefit from Pin 1 agonism or antagonism.

Further investigations are needed to explore the potential role of Pin1 as biomarker of neurodegeneration, in particular in the early stages of the disease. Based on the hypothesis that peripheral cells may allow to study in vitro the dynamic alterations of metabolic and biochemical processes that may reflect events occurring in the brain, future studies may help elucidating the possibility to measure Pin1 expression in easily accessible cells, such as peripheral tissues. In this regard, Ferri et al. reported a lower gene expression of Pin1 with a higher DNA methylation in three CpG sites at Pin1 gene promoter in FTD subjects, while a higher Pin1 gene expression with a lower DNA methylation in late-onset AD patients and controls, corroborating the hypothesis of a diverse involvement of Pin1 in different types of dementia [63].

## Data Availability

Not applicable.
